# Letter from the Editor in Chief

**DOI:** 10.19102/icrm.2023.14011

**Published:** 2023-01-15

**Authors:** Moussa Mansour


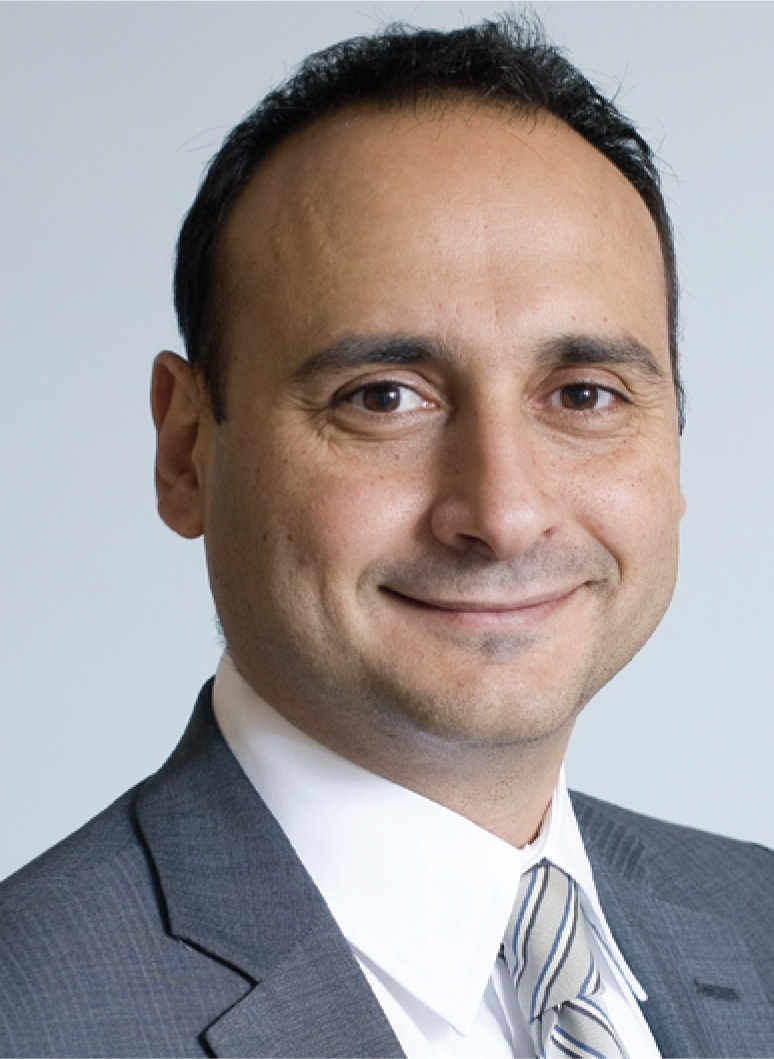


Dear readers,

Cardiac resynchronization therapy (CRT) is a primary treatment for patients with congestive heart failure (CHF) and conduction system disease. The benefit of this treatment modality has been demonstrated in many studies. Yet, despite many years of research and technological advances, a significant number of patients still do not experience improvement after CRT, and some studies estimate the percentage of non-responders to be >30%.

Over the years, different approaches have been used to improve the selectivity of the targeting areas in the left ventricle (LV) that would result in maximum resynchronization. One approach is electrical-based and aims to target areas of latest activation in the LV. Along this line, a novel technology, electrical dyssynchrony mapping, was recently described.^1^ It consists of acquiring a modified surface electrogram and generating dyssynchrony maps. Based on these maps, CRT optimization, including right and ventricular differential pacing, could be modified to improve electrical synchrony, which led to an improvement in LV ejection fraction. Another approach is mechanical-based and aims to pace areas of latest contraction in the LV. Identifying the areas of latest contraction has been conventionally guided by echocardiography. In this issue of *The Journal of Innovations in Cardiac Rhythm Management*, Abdellatif et al.^2^ describe the use of single-photon emission computed tomography for the identification of the areas of latest contraction and their proximity to the pacing lead to enable the prediction of CRT response.

Both electrical- and mechanical-based approaches to LV lead placement have led to only a small improvement in the rate of non-responders. The lack of a full response is probably due to the limitations imposed by the anatomy of the coronary venous tree rather than the knowledge of where to pace the LV. As a result, more focus should be directed toward the discovery of novel pacing technologies that overcome unsuitable coronary anatomies, including direct LV pacing, physiologic pacing, and others.


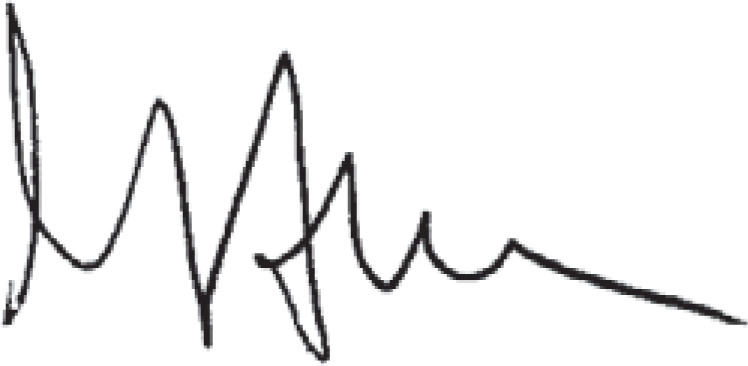


Sincerely,

Moussa Mansour, md, fhrs, facc

Editor in Chief

*The Journal of Innovations in Cardiac Rhythm Management*

MMansour@InnovationsInCRM.com

Director, Atrial Fibrillation Program

Jeremy Ruskin and Dan Starks Endowed Chair in Cardiology

Massachusetts General Hospital

Boston, MA 02114
